# Supplementary material for: The Addition of Glutamine Enhances the Quality of Huangjiu by Modifying the Assembly and Metabolic Activities of Microorganisms during the Fermentation Process
Source: Foods. 2024 Sep 6;13(17):2833. doi: 10.3390/foods13172833 (PMC11395270; doi:10.3390/foods13172833)
Supplement: Supplementary file 1 [file foods-13-02833-s001.zip › foods-3162356-supplementary.pdf]

Addition of glutamine enhances the quality of Huangjiu by modifying the assembly and metabolic activities of microorganisms during the fermentation process

Jia-Jia Jiang [a], Guan-Yu Fang [b,c,*], Chang-Ling Wu [b,c], Peng Wang [b,c], Yong-Zhu Zhang [b,c], Cheng Zhang [b,c], Feng-Hua Wu [c,d], Zhi-Chu Shan [e], Qing-Ru Liu [f], Xing-Quan Liu [a,b,c,*]

[a] College of Forestry and Biotechnology, Zhejiang A&F University, Hangzhou 311300, P.R. China

[b] College of Food & Health, Zhejiang A&F University, Hangzhou 311300, P.R. China

[c] National Grain Industry (High-Quality Rice Storage in Temperate and Humid Region) Technology Innovation Center, Zhejiang A&F University, Hangzhou 311300, P.R. China

[d] College of Advanced Agricultural Sciences, Zhejiang A&F University, Hangzhou 311300, P.R. China

[e] Zhejiang Pagoda Brand Shaoxing Rice Wine Co., Ltd., Shaoxing 312000, China

[f] Food Microbiology Key Laboratory of Sichuan Province, Xihua University, Chengdu 610039, Sichuan, China

*Corresponding author at: College of Food & Health, Zhejiang A&F University, Hangzhou 311300, P.R. China

E-mail address: fangguanyu@zafu.edu.cn (G.-Y. Fang) & liuxq@zafu.edu.cn (X.-Q. Liu)

Table S1. Content of volatile components at the end of fermentation (μg/L)

No.	Volatile components	CK	Glu	Gln	Asp	Asn
A1	Acetic acid, 2-methylpropyl ester	41.73±4.42^{ab}	42.12±0.26^{ab}	32.33±2.50^{b}	46.35±1.53^{a}	35.61±2.00^{ab}
A2	Butanoic acid, ethyl ester	12.05±1.44^{d}	29.73±1.08^{b}	23.74±1.73^{c}	23.78±2.85^{c}	37.00±0.41^{a}
A3	1-Butanol, 3-methyl-, acetate	345.28±74.41^{a}	318.45±19.39^{a}	215.10±18.89^{bc}	285.06±39.25^{ab}	185.95±22.69^{c}
A4	Pentanoic acid, ethyl ester	11.88±0.64^{b}	14.33±0.66^{a}	10.25±0.42^{b}	11.78±2.40^{b}	9.75±0.23^{b}
A5	Hexanoic acid, ethyl ester	273.92±26.56^{a}	254.06±30.09^{a}	191.19±9.31^{b}	191.06±24.45^{b}	180.22±10.81^{b}
A6	Acetic acid, hexyl ester	4.19±0.39^{c}	6.97±0.63^{a}	5.62±0.99^{b}	5.39±0.13^{b}	2.83±0.50^{d}
A7	Heptanoic acid, ethyl ester	31.65±6.92^{a}	28.81±0.77^{a}	16.05±0.29^{b}	13.46±3.64^{bc}	10.75±0.74^{c}
A8	Propanoic acid, 2-hydroxy-, ethyl ester	76.54±5.46^{ab}	69.39±4.37^{ab}	66.67±8.51^{b}	81.14±9.50^{a}	73.08±6.05^{ab}
A9	Isopentyl hexanoate	8.00±1.93	/	/	/	/
A10	Nonanoic acid, ethyl ester	63.45±8.54^{a}	58.03±22.22^{a}	15.82±3.99^{b}	/	/
A11	Decanoic acid, ethyl ester	375.97±33.65^{a}	279.31±27.37^{b}	129.97±8.79^{c}	139.42±27.95^{c}	40.85±3.53^{d}
A12	Acetic acid, 2-phenylethyl ester	84.22±11.07^{a}	54.83±5.76^{ab}	36.88±3.45^{ab}	40.85±1.01^{ab}	32.78±2.37^{b}
A13	2(3H)-Furanone, dihydro-5-pentyl-	14.22±0.94^{a}	12.72±0.34^{a}	/	/	/
A14	Nonanoic acid, 9-oxo-, methyl ester	163.74±2.89^{a}	73.21±2.17^{b}	58.26±1.62^{c}	38.39±0.84^{d}	29.80±3.27^{e}
A15	Isopropyl Myristate	18.25±1.20^{a}	/	10.65±0.23^{c}	/	14.08±1.09^{b}
A16	Tetradecanoic acid, ethyl ester	187.92±2.91^{b}	235.25±14.76^{a}	117.29±0.92^{c}	127.69±19.32^{c}	78.55±18.58^{d}
A17	Hexadecanoic acid, methyl ester	37.24±1.66^{a}	17.87±5.50^{b}	27.71±16.93^{ab}	/	35.75±1.64^{a}
A18	Hexadecanoic acid, ethyl ester	937.61±59.98^{b}	1121.51±60.94^{a}	642.68±12.64^{d}	806.20±85.45^{c}	576.29±81.58^{d}
A19	Octadecanoic acid, ethyl ester	16.46±1.94^{a}	16.11±0.52^{a}	/	/	/
A20	Methyl 8-oxooctanoate	/	11.97±0.17	/	/	/
A21	9-Octadecenoic acid (Z)-, methyl ester	/	37.24±4.56^{a}	/	27.70±0.16^{b}	/
A22	Butanedioic acid, diethyl ester	/	12.59±2.02^{a}	8.76±0.20^{b}	7.12±0.24^{b}	/
A23	E-11-Hexadecenoic acid, ethyl ester	/	23.41±0.87	/	/	/
A24	2,2,4-Trimethyl-1,3-pentanediol diisobutyrate	/	/	7.41±1.13^{a}	/	9.11±1.03^{a}
A25	Benzoic acid, ethyl ester	/	/	5.62±0.24^{a}	/	3.02±0.36^{b}
A26	10-Octadecenoic acid, methyl ester	/	/	31.98±3.95	/	/
A27	Dodecanoic acid, ethyl ester	/	/	/	/	20.98±0.74
A28	gamma-Decanolactone	/	/	/	/	12.04±1.31
	Total	2704.31±246.95^{a}	2717.92±204.46^{a}	1653.99±96.71^{c}	1845.38±218.71^{b}	1376.41±157.61^{d}
B1	1-Propanol, 2-methyl-	531.40±5.26^{b}	444.15±39.92^{d}	485.43±8.47^{c}	591.34±20.46^{a}	496.64±12.88^{c}
B2	1-Butanol, 3-methyl-	2499.58±240.37^{a}	2361.50±187.47^{a}	1827.32±235.46^{b}	2659.17±31.04^{a}	1936.82±328.19^{b}
B3	1-Hexanol	50.79±1.95^{ab}	56.23±3.39^{a}	45.06±0.35^{b}	54.78±6.17^{a}	50.50±1.94^{ab}
B4	2,3-Butanediol, [R-(R*,R*)]-	69.50±1.10^{bc}	77.76±11.27^{ab}	55.10±6.22^{c}	74.69±7.05^{ab}	87.73±12.53^{a}

B5	Silanediol, dimethyl-	145.73±9.33^{a}	107.56±23.50^{b}	77.75±8.03^{c}	/	71.70±10.62^{c}
B6	1-Nonanol	19.16±0.84^{a}	18.93±0.67^{a}	10.38±0.85^{c}	14.50±2.02^{b}	14.73±2.68^{b}
B7	Phenylethyl Alcohol	676.47±17.66^{a}	459.49±5.69^{b}	369.02±14.86^{d}	428.68±14.12^{c}	416.05±25.28^{c}
B8	1-Octanol	/	22.67±2.37	/	/	/
B9	1-Heptanol	/	13.84±1.12^{a}	7.79±0.03^{b}	/	8.11±0.92^{b}
B10	2-Nonanol	/	/	5.26±1.38^{a}	/	3.60±0.39^{a}
B11	2-Hexadecanol	/	/	8.03±0.81	/	/
B12	Ethanol, 2-(dodecyloxy)-	/	/	10.57±1.53^{a}	/	9.75±0.31^{a}
B13	1-Pentanol	/	/	2.76±0.45^{a}	2.92±0.21^{a}	/
B14	1-Propanol, 3-(methylthio)-	/	/	/	7.71±0.00^{a}	3.43±0.09^{b}
B15	E-11,13-Tetradecadien-1-ol	/	/	/	71.17±5.66	/
B16	2-Pentadecanol	/	/	/	5.74±0.66^{b}	11.79±1.40^{a}
B17	1-Propanol, 3-ethoxy-	/	/	/	/	3.87±0.65
B18	Cedrol	/	/	/	/	9.70±0.46
	Total	3992.63±276.51^{a}	3562.12±275.40^{ab}	2904.48±278.43^{c}	3910.71±87.39^{a}	3124.40±398.34^{bc}
C1	Acetic acid	647.71±221.53^{bc}	440.17±157.67^{c}	963.94±156.12^{a}	1000.31±231.83^{a}	856.79±39.50^{ab}
C2	Nonanoic acid	13.60±1.29^{a}	/	/	7.89±1.65^{b}	7.67±0.27^{b}
C3	Hexanoic acid	/	/	/	/	6.49±0.75
C4	Octanoic Acid	/	/	/	/	6.89±0.09
	Total	661.31±222.82^{bc}	440.17±157.67^{c}	963.94±156.12^{ab}	1008.20±233.48^{a}	877.84±40.62^{ab}
D1	Nonanal	13.36±2.22^{a}	9.95±0.62^{ab}	5.18±0.74^{b}	6.56±0.35^{ab}	8.78±0.23^{ab}
D2	Decanal	17.87±1.11^{a}	18.67±1.16^{a}	9.74±1.40^{c}	18.62±0.74^{a}	12.15±1.91^{b}
D3	Hexadecanal	/	/	/	/	8.32±0.11
	Total	31.23±3.33^{a}	28.62±1.78^{ab}	14.92±2.14^{c}	25.17±1.09^{b}	29.26±2.26^{ab}
E1	2-Octanone	10.47±1.39^{a}	10.14±1.02^{a}	11.55±2.25^{a}	9.62±1.42^{a}	12.62±2.12^{a}
E2	Benzofuran, 2,3-dihydro-	29.52±2.62^{a}	21.09±0.08^{b}	16.23±1.84^{c}	19.82±3.15^{bc}	22.89±1.59^{b}
E3	2-Methoxy-4-vinylphenol	116.83±9.54^{a}	83.58±5.21^{b}	79.01±4.44^{b}	64.92±7.91^{c}	78.28±3.46^{b}
E4	Phenol, 2,4-bis(1,1-dimethylethyl)-	807.87±69.55^{b}	/	/	731.40±27.17^{b}	1869.83±87.39^{a}
E5	Phenol, 3,5-bis(1,1-dimethylethyl)-	/	787.51±80.71	/	/	/
E6	Cyclohexene, 1-methyl-4-(1-methylethenyl)-, (S)-	17.88±1.44^{b}	6.78±1.22^{c}	/	29.67±8.58^{a}	/
E7	2-Tetradecene, (E)-	/	6.12±2.51^{b}	/	18.74±2.71^{a}	/
E8	5-Tetradecene, (E)-	/	/	6.16±0.20	/	/
E9	7-Tetradecene, (Z)-	/	/	/	46.75±10.61	/
	Total	1210.69±113.69^{b}	1040.74±101.14^{b}	249.99±25.96^{c}	933.43±62.91^{b}	2224.81±100.74^{a}

Table S2. Free amino acid content in the end of fermentation (mg/L)

Amino acid	CK	Glu	Gln	Asp	Asn
Asp	95.18±2.45^{a}	91.77±2.15^{b}	84.13±0.35^{c}	91.93±0.35^{b}	82.07±1.72^{c}
Glu	209.86±4.88^{a}	202.52±4.77^{b}	188.22±1.62^{c}	199.33±4.40^{b}	174.59±1.58^{d}
Ser	114.22±7.95^{b}	119.95±1.01^{b}	119.09±0.66^{b}	133.54±1.44^{a}	117.80±2.07^{b}
Gly	188.56±3.10^{b}	186.71±2.70^{bc}	177.78±0.39^{c}	201.92±0.26^{a}	179.10±10.86^{bc}
His	60.93±0.48^{d}	66.22±0.74^{c}	67.86±0.39^{bc}	71.19±0.65^{a}	69.38±2.01^{ab}
Thr	78.10±5.20^{b}	83.98±1.08^{b}	78.71±1.27^{b}	91.80±1.70^{a}	80.29±4.51^{b}
Arg	270.51±5.38^{a}	264.26±6.29^{a}	239.89±2.90^{b}	267.94±8.17^{a}	239.90±7.88^{b}
Ala	601.23±1.18^{b}	602.50±7.00^{b}	580.15±8.55^{c}	633.47±6.55^{a}	579.74±0.54^{c}
Pro	279.77±1.06^{b}	272.72±0.98^{c}	247.42±0.40^{d}	287.80±5.28^{a}	240.70±3.87^{e}
Tyr	295.46±1.47^{c}	303.93±5.65^{b}	300.60±2.17^{bc}	333.85±4.05^{a}	299.01±4.98^{bc}
Val	231.38±6.50^{d}	240.95±3.94^{bc}	236.44±2.42^{cd}	264.50±2.40^{a}	245.64±0.67^{b}
Met	105.30±5.92^{b}	111.47±1.04^{b}	108.92±2.30^{b}	119.36±2.79^{a}	105.65±2.69^{b}
Ile	43.39±2.13^{c}	48.36±0.76^{ab}	46.15±1.99^{bc}	50.46±1.44^{a}	46.57±0.67^{b}
Leu	152.74±8.03^{c}	164.76±4.26^{b}	166.59±4.47^{ab}	175.61±2.96^{a}	164.19±4.52^{b}
Cys	380.09±7.44^{c}	399.68±6.51^{b}	391.48±1.87^{bc}	449.47±3.36^{a}	394.91±11.21^{b}
Phe	344.80±8.13^{b}	355.26±5.70^{b}	354.03±8.51^{b}	398.29±5.41^{a}	358.20±6.06^{b}
Lys	223.96±2.61^{d}	251.29±3.99^{c}	258.40±6.57^{bc}	281.85±5.29^{a}	262.61±2.31^{b}
Total	3675.48±57.91^{c}	3766.33±28.56^{b}	3645.87±24.27^{c}	4052.34±12.69^{a}	3640.33±46.10^{c}

Table S3. Effect of Gln addition on volatile components (μg/L)

No.	Volatile components	CK	Glu-200	Glu-400
A1	Acetic acid, pentyl ester	513.72±56.14[ab]	584.04±30.06[a]	467.82±20.88[b]
A2	Pentanoic acid, ethyl ester	28.04±1.75[b]	31.92±1.00[a]	32.00±1.82[a]
A3	Hexanoic acid, ethyl ester	624.02±16.64[a]	617.32±10.69[a]	627.48±11.63[a]
A4	Acetic acid, hexyl ester	7.47±1.34[b]	5.54±0.27[b]	9.52±1.07[a]
A5	Propanoic acid, 2-hydroxy-, ethyl ester	161.83±31.30[a]	36.19±1.09[c]	80.94±1.57[b]
A6	Heptanoic acid, ethyl ester	86.81±5.32[a]	82.07±6.06[a]	77.86±5.89[a]
A7	Octanoic acid, ethyl ester	402.51±29.00[a]	358.05±30.09[a]	374.19±43.90[a]
A8	Isopentyl hexanoate	5.73±0.42[b]	7.38±0.05[a]	/
A9	Isoamyl lactate	4.40±1.04[b]	6.33±1.44[ab]	7.14±0.42[a]
A10	Nonanoic acid, ethyl ester	59.58±5.20[a]	39.68±0.21[b]	42.20±6.38[b]
A11	Butanedioic acid, diethyl ester	6.30±1.31[a]	6.02±0.58[ab]	4.38±0.32[b]
A12	Decanoic acid, ethyl ester	144.50±3.36[ab]	123.18±20.61[b]	157.39±17.82[a]
A13	4-Decenoic acid, ethyl ester, (Z)-	5.44±0.84	/	/
A14	Acetic acid, 2-phenylethyl ester	54.59±1.62[a]	40.19±0.29[b]	40.88±8.85[b]
A15	Dodecanoic acid, ethyl ester	28.87±0.23[b]	40.32±2.91[a]	25.51±2.78[b]
A16	2(3H)-Furanone, dihydro-5-pentyl-	17.33±0.93[b]	17.97±1.28[ab]	20.69±2.20[a]
A17	Tetradecanoic acid, ethyl ester	111.03±17.18[a]	54.20±5.29[b]	/
A18	Hexadecanoic acid, methyl ester	42.54±4.93[a]	/	30.53±5.53[b]
A19	Diethyl Phthalate	44.94±2.24[a]	38.09±0.86[b]	38.77±1.89[b]
A20	Hexadecanoic acid, ethyl ester	374.89±14.99[a]	231.69±22.63[b]	249.96±18.25[b]
A21	1,2-Benzenedicarboxylic acid, bis(2-methylpropyl) ester	20.46±1.50[a]	/	25.34±6.20[a]
A22	(E)-9-Octadecenoic acid ethyl ester	131.38±17.06[a]	93.31±4.58[b]	120.57±23.61[ab]
A23	Benzoic acid, ethyl ester	3.53±0.03[a]	3.23±0.09[a]	3.77±0.97[a]
A24	Ethyl tridecanoate	107.10±21.31[a]	/	117.75±7.36[a]
A25	9-Octadecenoic acid (Z)-, methyl ester	88.44±42.31[a]	/	36.66±9.80[b]
A26	9,12-Octadecadienoic acid, methyl ester	17.74±0.88	/	/
A27	Linoleic acid ethyl ester	130.59±11.39[a]	49.56±7.85[b]	96.21±19.09[b]
A28	Benzeneacetic acid, ethyl ester	/	3.52±0.57	/
A29	Oxalic acid, 2-ethylhexyl pentyl ester	/	/	16.95±1.63
	Total	3223.77±26.28[a]	2469.81±54.18[c]	2704.53±64.95[b]
B1	1-Propanol, 2-methyl-	501.33±40.92[b]	697.22±60.93[a]	630.06±99.37[ba]
B2	1-Butanol, 3-methyl-	2430.98±206.95[c]	3820.18±106.63[a]	3085.77±317.68[b]
B3	2-Heptanol	4.47±0.41[a]	3.69±0.10[ab]	3.41±0.56[b]
B4	1-Hexanol	56.56±2.18[a]	55.50±3.13[a]	59.59±3.11[a]
B5	1-Octen-3-ol	13.75±0.84[a]	13.36±1.55[a]	14.83±1.50[a]
B6	1-Heptanol	14.03±1.04[b]	16.08±0.49[a]	16.15±0.39[a]
B7	1-Octanol	25.21±1.90[a]	27.92±1.60[a]	25.87±2.67[a]
B8	1-Nonanol	12.67±0.69[c]	16.41±0.14[a]	14.75±1.05[b]
B9	Phenylethyl Alcohol	490.95±3.73[b]	585.97±3.66[a]	540.74±46.08[ab]
B10	1-Butanol	5.77±0.18[b]	8.57±0.44[a]	9.12±0.66[a]
B11	2,3-Butanediol	44.28±1.06[b]	52.77±0.63[a]	46.14±1.21[b]

B12	Silanediol, dimethyl-	21.13±0.50^{b}	24.01±0.83^{a}	24.33±0.00^{a}
B13	1-Octanol, 2-butyl-	8.28±0.01^{b}	11.78±0.53^{a}	10.48±1.96^{ab}
B14	2-Tetradecanol	7.72±1.20	/	/
B15	6-Octen-1-ol, 3,7-dimethyl-	/	3.72±1.13	/
B16	2-Heptanol, (S)-	/	4.81±0.37	/
B17	Ethanol, 2-(dodecyloxy)-	/	/	12.36±5.50
B18	1-Dodecanol	/	/	12.24±4.77
	Total	3637.14±222.72^{c}	5341.99±176.03^{a}	4505.84±271.55^{b}
C1	Acetic acid	435.77±66.63^{a}	276.84±55.26^{b}	441.46±35.27^{a}
C2	Propanoic acid, 2-methyl-	13.64±3.51^{b}	23.36±1.20^{a}	22.34±4.17^{a}
C3	Hexanoic acid	9.89±1.19^{b}	11.22±1.13^{ab}	14.80±3.37^{a}
C4	Octanoic Acid	15.53±0.32^{b}	18.23±0.46^{a}	16.10±0.83^{b}
C5	Nonanoic acid	5.22±2.03^{a}	/	5.77±0.45^{a}
C6	n-Decanoic acid	14.68±0.31^{a}	14.92±1.25^{a}	13.95±0.47^{a}
C7	Butanoic acid	/	10.70±6.67	/
	Total	494.73±70.97^{a}	355.28±65.63^{b}	514.41±28.50^{a}
D1	Decanal	6.08±0.63^{b}	14.50±1.64^{a}	8.38±2.54^{b}
D2	Benzaldehyde, 2,4-dimethyl-	16.40±2.48^{a}	16.24±3.38^{a}	17.93±2.12^{a}
	Total	22.48±1.85^{b}	30.74±2.89^{a}	26.30±0.42^{b}
E1	2-Octanone	10.70±0.51^{b}	14.49±2.19^{a}	12.66±0.34^{ab}
E2	2-Decanone	/	6.27±1.01^{a}	6.39±0.60^{a}
E3	2-Nonanone	/	4.22±0.73^{a}	2.37±0.06^{b}
	Total	10.70±0.51^{c}	24.98±2.60^{a}	21.41±0.92^{b}
F1	2-Methoxy-4-vinylphenol	36.80±4.94^{c}	70.79±2.55^{a}	51.22±0.96^{b}
F2	Limonene	/	27.31±2.38	/
F3	Phenol, 2,4-bis(1,1-dimethylethyl)-	/	15.58±1.10^{a}	11.48±4.10^{a}
	Total	36.80±4.94^{c}	113.68±1.27^{a}	62.70±3.35^{b}

Table S4. Effect of Gln addition on free amino acids (mg/L)

Amino acid	CK	Gln-200	Gln-400
Asp	74.58±3.30[a]	76.15±2.44[a]	78.94±0.97[a]
Glu	143.15±0.86[a]	145.71±23.65[a]	121.78±1.27[a]
Umami amino acids	217.73±3.03[a]	221.86±21.60[a]	200.72±2.24[a]
Ser	88.55±3.27[a]	92.35±0.89[a]	92.66±1.21[a]
Gly	150.88±3.78[b]	178.81±12.74[a]	175.44±9.19[a]
Thr	86.98±2.62[c]	96.21±3.16[b]	101.92±2.48[a]
Ala	212.31±3.90[a]	264.80±6.94[b]	216.95±11.95[a]
Pro	166.34±6.01[c]	201.51±4.84[b]	212.86±3.46[a]
Sweet amino acids	705.06±7.86[c]	833.67±20.48[a]	799.84±14.77[b]
His	76.74±4.51[a]	78.46±1.19[a]	74.49±5.17[a]
Arg	580.27±6.78[b]	717.43±19.20[a]	721.26±15.78[a]
Tyr	299.06±8.29[b]	317.04±6.04[a]	311.08±6.69[ab]
Val	226.57±6.64	223.43±4.40	221.32±0.62
Ile	109.35±4.65[b]	120.25±1.34[a]	116.14±0.96[a]
Leu	322.87±12.50[b]	357.08±6.22[a]	369.47±2.09[a]
Phe	311.64±6.18[b]	329.92±2.71[a]	337.44±2.22[a]
Lys	282.33±10.83[b]	309.10±2.35[a]	314.21±4.93[a]
Bitter amino acids	2208.82±10.25[b]	2452.71±32.33[a]	2465.41±36.06[a]
Met	82.94±0.08[b]	84.63±2.42[b]	95.51±1.37[a]
Cys	81.72±4.32[a]	71.96±2.20[b]	76.14±0.46[ab]
Salty amino acids	164.66±4.40[b]	156.59±1.31[c]	171.65±1.30[a]
Total	3296.27±5.77[b]	3664.83±34.03[a]	3637.62±53.12[a]

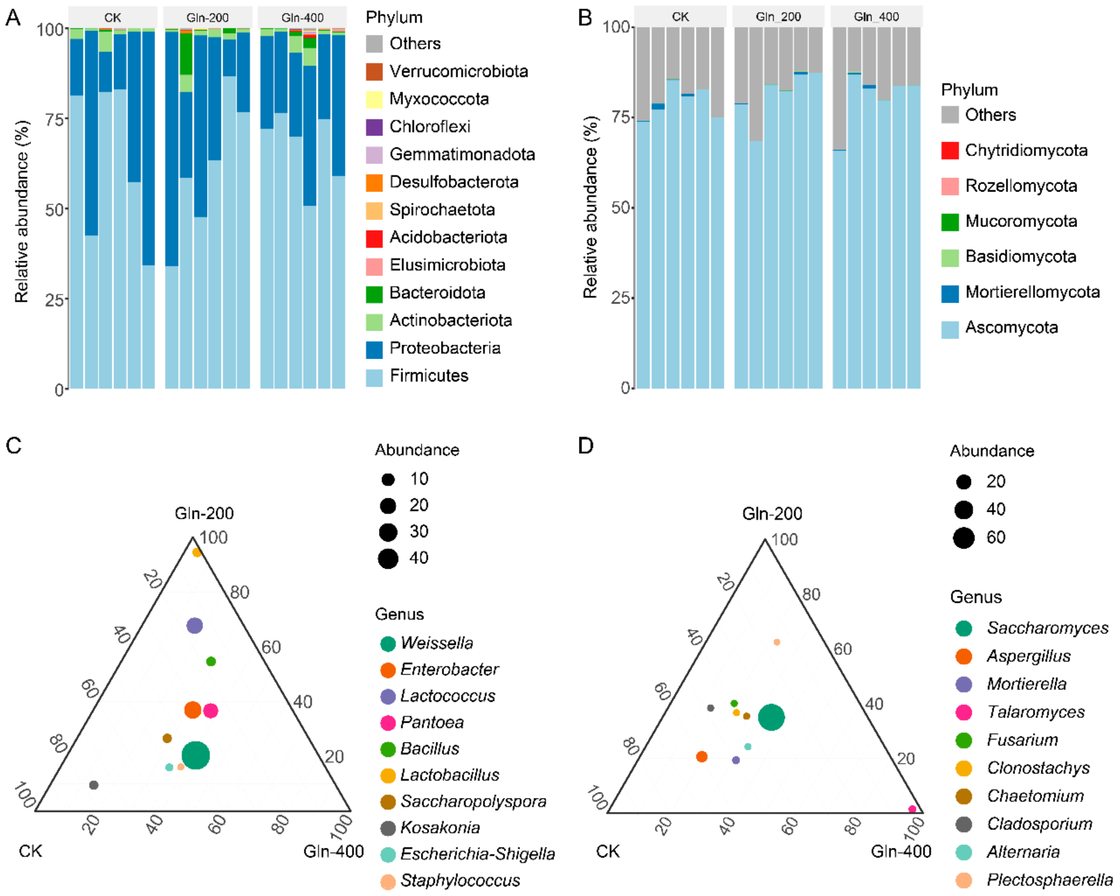

Fig. S1. Relative abundance of bacteria (A) and fungi (B) at the phylum level. Relative abundance of bacteria (C) and fungi (D) at the genus level.
